# Experimental Evidence of A_2A_–D_2_ Receptor–Receptor Interactions in the Rat and Human Carotid Body

**DOI:** 10.3389/fphys.2021.645723

**Published:** 2021-04-15

**Authors:** Elena Stocco, Maria Martina Sfriso, Giulia Borile, Martina Contran, Silvia Barbon, Filippo Romanato, Veronica Macchi, Diego Guidolin, Raffaele De Caro, Andrea Porzionato

**Affiliations:** ^1^Department of Neuroscience, Institute of Human Anatomy, University of Padova, Padua, Italy; ^2^Department of Physics and Astronomy “G. Galilei,” University of Padova, Padua, Italy; ^3^Institute of Pediatric Research Città della Speranza, Padua, Italy

**Keywords:** carotid body, type I cells, adenosine A_2A_ receptors, dopamine D_2_ receptors, heterodimes, *in situ* PLA

## Abstract

Adenosine A_2A_ receptors (A_2A_R) and dopamine D_2_ receptors (D_2_R) are known to be involved in the physiological response to hypoxia, and their expression/activity may be modulated by chronic sustained or intermittent hypoxia. To date, A_2A_R and D_2_R can form transient physical receptor–receptor interactions (RRIs) giving rise to a dynamic equilibrium able to influence ligand binding and signaling, as demonstrated in different native tissues and transfected mammalian cell systems. Given the presence of A_2A_R and D_2_R in type I cells, type II cells, and afferent nerve terminals of the carotid body (CB), the aim of this work was to demonstrate here, for the first time, the existence of A_2A_R–D_2_R heterodimers by *in situ* proximity ligation assay (PLA). Our data by PLA analysis and tyrosine hydroxylase/S100 colocalization indicated the formation of A_2A_R–D_2_R heterodimers in type I and II cells of the CB; the presence of A_2A_R–D_2_R heterodimers also in afferent terminals is also suggested by PLA signal distribution. RRIs could play a role in CB dynamic modifications and plasticity in response to development/aging and environmental stimuli, including chronic intermittent/sustained hypoxia. Exploring other RRIs will allow for a broad comprehension of the regulative mechanisms these interactions preside over, with also possible clinical implications.

## Introduction

In mammals and humans, the carotid bodies (CBs) are chemosensory organs located at the bifurcations of the common carotid arteries with a critical role in maintaining homeostasis during both development/aging (Di Giulio, 2018; Sacramento et al., 2019) and environmental variations (e.g., levels of O_2_, CO_2_, and arterial blood pH) (Iturriaga and Alcayaga, 2004; Iturriaga et al., 2016; Prabhakar and Peng, 2017; Di Giulio, 2018; Iturriaga, 2018) with also a sensing function with respect to metabolic factors (Porzionato et al., 2011; Conde et al., 2018; Cunha-Guimaraes et al., 2020; Sacramento et al., 2020).

The CB regulatory function is strictly related to its specific organization. Morphologically, in the CB parenchyma two types of cells can be distinguished: “neuron-like” chemosensitive type I cells, positive for tyrosine hydroxylase (TH), and “glial-like” supportive type II cells, positive for glial fibrillary acidic protein (GFAP) (Pardal et al., 2007; Tse et al., 2012). Sensitive innervation of the CB is mainly mediated by afferent terminals of the carotid sinus nerve, branch of the glossopharyngeal nerve, arising from neurons located in the petrosal ganglion (PG).

Neurotransmission in the CB involves a complex interplay of excitatory and inhibitory signals (Iturriaga and Alcayaga, 2004; Nurse, 2005; Fitzgerald et al., 2009; Porzionato et al., 2018; Stocco et al., 2020). Type I cells produce several neurotransmitters [e.g., dopamine, noradrenaline, adrenaline, acetylcholine, serotonin, adenosine, adenosine 5′-triphosphate (ATP)] and neuromodulators (e.g., enkephalins, neuropeptide Y, calcitonin gene-related peptide, galanin, endothelins, bombesin, adrenomedullin, kisspeptins, leptin) (Varas et al., 2003; Iturriaga and Alcayaga, 2004; Porzionato et al., 2008), in turn acting in an autocrine/paracrine manner on a broad spectrum of different ionotropic/metabotropic receptors located in afferent nerve fibers, type I cells, and type II cells, these latter also showing a role in the coordination of chemosensory transduction (Nurse, 2014; Porzionato et al., 2018; Stocco et al., 2020). Among these receptors, some metabotropic G protein-coupled receptors (GPCRs) (e.g., A_2A_, D_1__/__2_, H_1__/__2__/__3_, M_1__/__2_, 5-HT_2A_, and others) are also involved; in particular, A_2A_ and D_2_ have attracted the attention of many researchers, resulting among the most studied GPCRs (Aldossary et al., 2020).

The presence of A_2A_R was verified in rat (Gauda, 2000; Kobayashi et al., 2000; Xu et al., 2006; Bairam et al., 2009) and human (Fagerlund et al., 2010) CB specimens, where it showed to be expressed in type I cells, colocalizing with tyrosine-hydroxylase (TH) (Gauda, 2000; Gauda et al., 2000; Kobayashi et al., 2000; Bairam et al., 2009) or β-III-tubulin (Fagerlund et al., 2010). Considering the methodological approaches, different techniques were adopted, including *in situ* hybridization analysis (Gauda, 2000), immunohistochemistry (Kobayashi et al., 2000; Fagerlund et al., 2010), Western blot analysis (Bairam et al., 2009), and Ca^2+^ imaging technique (Xu et al., 2006). Also, D_2_R presence was reported in CB type I cells in rats (Czyzyk-Krzeska et al., 1992; Holgert et al., 1995; Gauda et al., 1996, 2001; Bairam and Khandjian, 1997; Gauda, 2000; Kinkead et al., 2005; Wakai et al., 2015), rabbits (Bairam et al., 1996b; Bairam and Khandjian, 1997; Bairam et al., 2003), cats (Bairam and Khandjian, 1997), and humans (Fagerlund et al., 2010). CB specimens were analyzed through *in situ* hybridization (Czyzyk-Krzeska et al., 1992; Holgert et al., 1995; Gauda, 2000), RT-PCR (Bairam et al., 1996b, 2003; Bairam and Khandjian, 1997; Kinkead et al., 2005), and immunofluorescence (Wakai et al., 2015).

Apart from type I cells, some data support the expression of A_2A_R and D_2_R also in type II cells (Kaelin-Lang et al., 1998; Leonard and Nurse, 2020). Additionally, A_2A_R (Gauda, 2000; Gauda et al., 2000; Conde et al., 2006, 2017, 2012; Zhang et al., 2018; Sacramento et al., 2019) and D_2_R (Czyzyk-Krzeska et al., 1992; Schamel and Verna, 1993; Bairam et al., 1996a, b) were also demonstrated in PG neurons and afferent fibers in the CB.

As demonstrated for transfected mammalian cell systems and different native tissues (i.e., central nervous system, mammary gland, liver, cancer tissues), A_2A_R and D_2_R can establish transient physical receptor–receptor interactions (RRIs) giving rise to a dynamic equilibrium between their specific monomeric form and homo/heterocomplexes (dimers or receptor mosaics) (Ferré et al., 2014; Guidolin et al., 2015). Such RRIs, in turn, likely modulate ligand binding and signaling, thus affecting the physio-pathological features but also the pharmacology of the nervous system.

Despite that the presence of A_2A_R and D_2_R has been broadly recognized in the CB, the possible existence of A_2A_R–D_2_R heterodimers was never verified before, but only hypothesized in a previous work (Porzionato et al., 2018). Thus, in this study, rat and human CB specimens were investigated by proximity ligation assay (PLA) technique to assess the eventual interaction between A_2A_R and D_2_R, thus corroborating the above working hypothesis and possibly opening the doors to the analysis of further possible RRIs in the CB.

## Materials and Methods

### Tissue Collection

The animal study was reviewed and approved by the ethical committee of Padua University, in agreement with the Italian Department of Health guidelines (Authorization No. 702/2016-PR of July 15, 2016). Human tissues were managed by the Body Donation Program of the Section of Human Anatomy, University of Padova (Macchi et al., 2011; Porzionato et al., 2012), according to European, Italian, and Regional guidelines (De Caro et al., 2009; Riederer et al., 2012). Excision was further authorized by the Italian law No. 10 of February 10, 2020, entitled “Rules regarding the disposition of one’s body and post-mortem tissues for study, training, and scientific research purposes” (Boscolo-Berto et al., 2020). Donors’ written informed consent was signed upon joining the Body Donation Program; here, Donor’s authorization expressly allowed to use Body and Body Parts also for research purposes, after donation.

Rat CBs were excised from 5 adult Sprague-Dawley rats; tissue isolation occurred immediately after euthanasia. Human CBs were obtained at autopsy from 5 adult subjects [3 males, 2 females; mean age 46 years, standard deviation (SD) ± 3.6] with no clinical sign of chronic pulmonary and/or cardiovascular diseases. Eventual pharmacological therapies that could have influenced the CB plasticity constituted a further exclusion criterion. Autopsies occurred within 30 h after death, according to Italian Law. On the basis of our previous experience (Porzionato et al., 2005, 2006, 2011), the tissues are viable and adequate for immunohistochemistry/immunofluorescence studies after this death–autopsy interval.

According to routine protocols, once isolated, the CBs were promptly fixed in 10% phosphate-buffered formalin for 72 h, dehydrated through ascending alcohols and xylene, clarified through xylene, and paraffin embedded.

### Immunohistochemical Analysis

Preliminarily, the primary antibodies used were tested by immunohistochemistry; this is an important step before PLA assay, whose performance critically depends on the antibodies’ quality as the GPCR antibodies are notoriously problematic (Michel et al., 2009; Trifilieff et al., 2011).

Longitudinal serial sections of the whole fixed carotid bifurcation (5 μm in thickness) were prepared, dewaxed according to routine protocols, and immunostained by anti-A_2A_R antibody (monoclonal mouse antibody; ab79714, Abcam, United Kingdom) and anti-D_2_R antibody (polyclonal rabbit antibody; ab150532, Abcam). The anti-A_2A_R antibody and the anti-D_2_R antibody were used with a dilution of 1:100 and 1:200, respectively; antigen retrieval occurred before both staining with high pH (EnVision^TM^ FLEX, High pH, K8012) and low pH (EnVision^TM^ FLEX, Low pH, K8005) buffer. The sections were incubated using the DAKO Autostainer Plus Staining System (EnVision^TM^ FLEX, High pH). Immunostaining specificity was confirmed by sections incubated without primary antibody, which did not show immunoreactivity.

### Proximity-Ligation Assay (PLA)

PLA technology allows easy visualization of endogenous protein–protein interactions at the single-molecule level. The method relies on the use of combinations of antibodies coupled to complementary oligonucleotides that are amplified and revealed with a fluorescent probe. Each single protein–protein interaction is visualized as a fluorescent spot.

*In situ* PLA was performed according to the manufacturer’s instructions on 5-μm rat and human CB slices using the following: mouse anti-A_2A_ primary antibody (dilution: 1:100); rabbit anti-D_2_R primary antibody (dilution: 1:200); Duolink^®^ *in situ* PLA detection kit (DUO92014, Sigma-Aldrich, St Louis, MO, United States); Duolink^®^ anti-rabbit PLUS probe (DUO92002, Sigma-Aldrich); and Duolink^®^ anti-mouse MINUS probe (DUO82040, Sigma-Aldrich). Briefly, the slices were blocked with Duolink^®^ blocking solution, in a humid chamber for 60 min at 37°C and then incubated with the primary antibodies (anti-A_2A_R and anti-D_2_R) solution prepared in the antibody diluent solution; incubation occurred in a humid chamber for 1 h at room temperature (RT). Thereafter, the primary antibody solution was tapped off and the slices were washed with wash buffer at RT, before incubation with the anti-rabbit and anti-mouse secondary antibody-conjugated PLA probes in a preheated humidity chamber, for 1 h at 37°C. After hybridization, ligation and amplification steps were performed. For TH and S100 colocalization analysis, after the amplification step, the slices were rinsed in wash buffer and (a) incubated with anti-TH (1:6,000) in Antibody Diluent solution (Dako) or (b) incubated with anti-S100 (1:7,000) in a humid chamber at 4°C, overnight. Subsequently, after a wash in PBS, incubation was performed using mouse Alexa Fluor-488 (1:100; 1 h at RT) for TH or rabbit Alexa Fluor-488 (1:500; 1 h at RT) for S100. Thereafter, the sections were rinsed in PBS and mounted with Vectashield mounting medium for fluorescence with DAPI (Vector Laboratories, Burlingame, CA, United States) for 15 min at RT.

Immunofluorescence and PLA signals were analyzed and acquired with Zeiss800 confocal microscope equipped with 63× oil objective (NA = 1.4). For each field of view, z-stacks were acquired for a total thickness of 10 μm. Images were acquired enabling the identification of the A_2A_R–D_2_R heterodimers at confocal microscopy as red dots. In order to better detail the localization of the red dots with reference to nuclei and membranes of different cell types, images were analyzed with the help of z projections and 3D volume rendering through different perspectives. This permitted to better localize the red dots without bias due to plane overlapping. In particular, Z-stacks were acquired and exported with ZenBlue software. For image visualization in 2D, z projections were performed with FIJI software, while 3D volume rendering was reconstructed with IMARIS software.

Negative control experiments were performed avoiding the conjugation of the primary antibodies with the Duolink^®^ Probes; in turn, no positive reaction occurred, and no red dots were visualized. The specificity of the double immunolabeling was verified by replacing the primary antibodies with PBS.

Quantification of the PLA signal was performed on z-stack images, and the number of red dots was manually counted with ImageJ, using the cell counter plugin. The average data referring to the density of positive PLA elements ± SD are related to images acquired from at least 3 randomly chosen fields from three slides of each animal/patient. The percentages of colocalization of red dots with TH and S100 immunostainings were also calculated.

## Results

### A_2A_R and D_2_R by Immunohistochemistry

**FIGURE 1:**
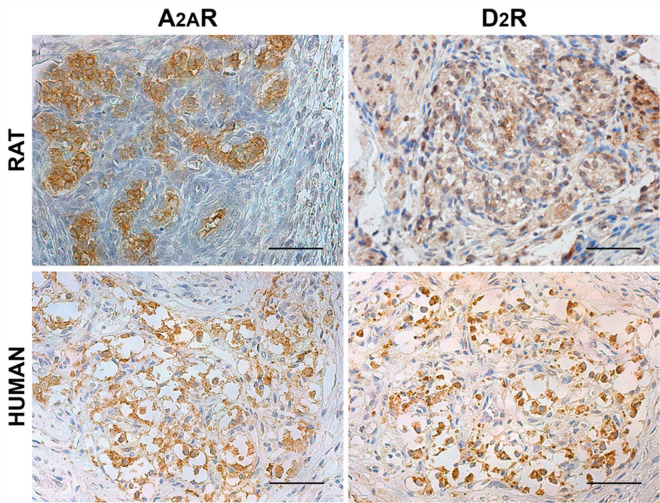
Distribution of adenosine A_2A_R and dopamine D_2_R in rat and human CB. Positive immunostaining in clusters of roundish cells is consistent with type I cells, although some positive elongated cells may be considered as type II cells. Scale bar: 50 μm.

Adenosine A_2A_R and dopamine D_2_R were identified through immunohistochemistry in both rat and human CBs (Figure 1). Considering 3′-diaminobenzidine tetrahydrochloride (DAB) distribution, A_2A_R- and D_2_R-positive elements were mainly localized in correspondence of type I cells, being similarly arranged in clusters. However, partial immunostaining of A_2A_R and D_2_R also in type II cells and PG nerve terminals cannot be excluded.

### Detection of the A_2A_R–D_2_R Heterodimers by PLA

**FIGURE 2:**
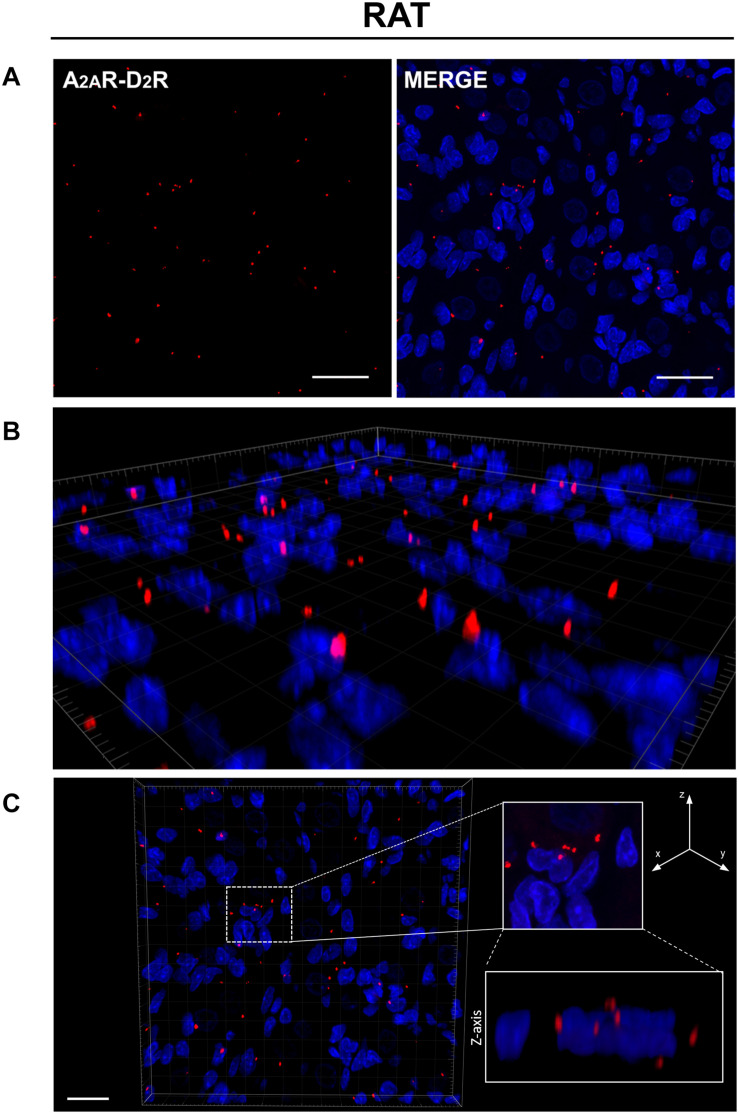
**(A)** Evidence for the existence of A_2A_R–D_2_R heterodimers in rat CB samples by PLA. Red dots showed the proximity of adenosine A_2A_R and dopamine D_2_R, indicating A_2A_R–D_2_R heterodimerization. The merged images highlighted the A_2A_R–D_2_R localization with respect to the cell nuclei (blue-fluorescent DAPI). Scale bar: 25 μm. **(B)** Representative 3D volume rendering of a sample area from **(A)** allowing assessment of red dot localization with respect to the cell nuclei. **(C)** Representative image of nuclei/red dots apparently appearing as superimposed (white dotted square in the image; corresponding magnification on the right side insert) and visualized in detail through z projection (lower right insert), thus showing a localization adjacent to the nucleus, but not inside it. Scale bar: 20 μm.

**FIGURE 3:**
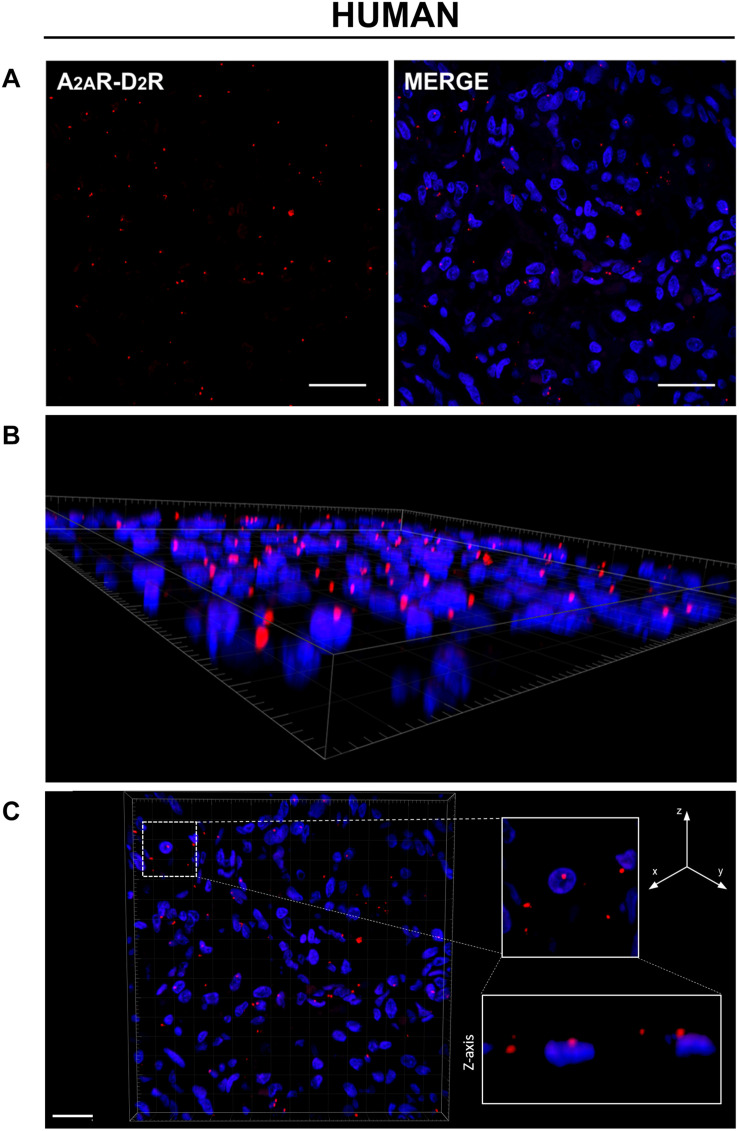
**(A)** Evidence for the existence of A_2A_R–D_2_R heterodimers in human CB samples by PLA. Red dots showed the proximity of adenosine A_2A_R and dopamine D_2_R, indicating A_2A_R–D_2_R heterodimerization. The merged images highlighted the A_2A_R–D_2_R localization with respect to the cell nuclei (blue-fluorescent DAPI). Scale bar: 25 μm. **(B)** Representative 3D volume rendering of a sample area from **(A)** allowing assessment of red dot localization with respect to the cell nuclei. **(C)** Representative image of nuclei/red dots apparently appearing as superimposed (white dotted square in the image; corresponding magnification on the right side insert) and visualized in detail through z projection (lower right insert), thus showing a localization adjacent to the nucleus, but not inside it. Scale bar: 20 μm.

PLA is an antibody-based method to detect biomolecules in physical proximity, and thus, it is recognized as an important experimental approach to demonstrate physical RRIs when native molecules are localized within a radius of 0–16 nm, a distance considered crucial for heteromer formation. Only in case of physical closeness of proteins will a signal be produced. Here, possible heterodimerization between A_2A_R and D_2_R was verified through PLA. Under a confocal microscope, in both rat and human CBs, most red dots were recognized close to the DAPI-stained nuclei but not inside them, supporting location at the plasma membrane level; 3D visualization of the tissues allowed for a better topographical analysis (Figures 2, 3). As it regards the few red dots which were far away from the nuclei, we cannot exclude a localization in afferent terminals from the carotid sinus nerve. A homogeneous distribution of heterodimers was observed in all specimens with a mean density (± standard deviation) of (3.5 ± 0.67) × 10^–3^ heterodimers/μm^2^ and (5.9 ± 1.4) × 10^–3^ heterodimers/μm^2^ in rat and human samples, respectively.

**FIGURE 4:**
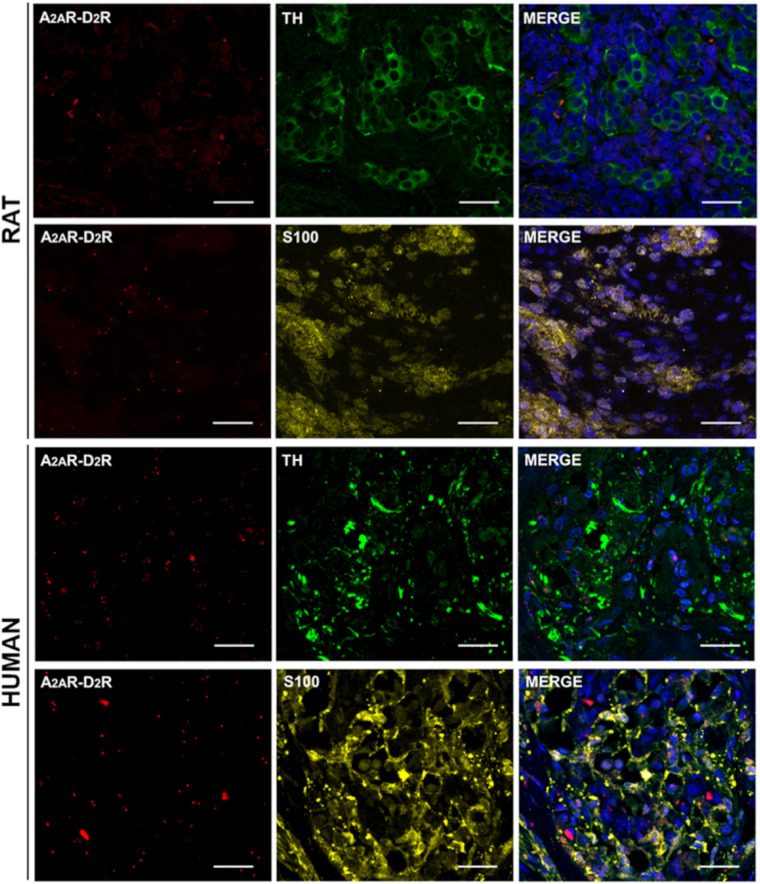
TH and S100 immunofluorescence in representative rat and human CB samples after A_2A_R–D_2_R PLA. Red dots showed A_2A_–D_2_ receptors heterodimers; anti-TH (visualized in green) and anti-S100 (visualized in yellow) staining allowed to distinguish type I and type II cells, respectively. Cell nuclei were recognizable after DAPI staining (visualized in blue) in the merge images. Scale bar: 25 μm.

After PLA, specific TH and S100 immunostaining was also performed to distinguish type I and type II cells. This methodological approach allowed to assess by confocal microscopy the localization of the A_2A_R–D_2_R heterodimers with respect to the CB constituent cells. In all stained specimens, S100-immunopositive cells, corresponding to type II cells, were specifically visualized as yellow elements; TH immunoreactivity was observed in the cytoplasm of CB type I cells and visualized in green (Figure 4). We cannot exclude possible visualization of TH-positive afferent nerve fibers, as also PG terminals may be TH immunoreactive (Katz et al., 1983; Katz and Black, 1986).

In rats, the percentages of TH and S100 colocalizations of red dots were 51.49 ± 5.97 and 55.84 ± 3.63, respectively. In human samples, the percentages of TH and S100 colocalizations of red dots were 60.65 ± 8.49 and 50.75 ± 6.30, respectively. Significant differences were not found between TH and S100 colocalizations in the two different species.

As previously stated, the presence of red dots far away from nuclei and possibly positive for TH may be interpreted as localization of A_2A_R–D_2_R heterodimers in PG terminals, as they may express A_2A_R (Gauda, 2000; Gauda et al., 2000; Conde et al., 2012; Zhang et al., 2018; Sacramento et al., 2019) and D_2_R (Czyzyk-Krzeska et al., 1992; Schamel and Verna, 1993; Bairam et al., 1996a, b).

## Discussion

Type I CB cells release many different neurotransmitters (i.e., acetylcholine, adenosine, ATP, dopamine) with excitatory or inhibitory effects (Iturriaga and Alcayaga, 2004). Among the main receptors mediating adenosine and dopamine function in the CB, A_2A_R, and D_2_R are included. Experimental data support the presence of A_2A_R (Gauda, 2000; Gauda et al., 2000; Kobayashi et al., 2000; Xu et al., 2006; Bairam et al., 2009; Fagerlund et al., 2010) and D_2_R (Czyzyk-Krzeska et al., 1992; Holgert et al., 1995; Gauda et al., 1996, 2001; Bairam et al., 1996b, 2003; Bairam and Khandjian, 1997; Gauda, 2000; Kinkead et al., 2005; Fagerlund et al., 2010; Wakai et al., 2015) in CB type I cells. Kaelin-Lang et al. (1998) also recognized, by *in situ* hybridization on rats, A_2A_R-positive elements likely attributable to type II cells. Even referring to D_2_R, a recent paper by Leonard and Nurse (2020), considering the possible inhibitory role of dopamine on type II cell function, suggests D_2_R localization in type II cells. Our data indicated the presence of A_2A_R–D_2_R heterodimers both in type I and II cells of rats and humans.

PG neurons and terminals are known to express A_2A_R (Gauda, 2000; Gauda et al., 2000; Conde et al., 2012; Zhang et al., 2018; Sacramento et al., 2019) and D_2_R (Czyzyk-Krzeska et al., 1992; Schamel and Verna, 1993; Bairam et al., 1996a, b). Moreover, afferent terminals in the CB may also express TH (Katz et al., 1983; Katz and Black, 1986). Thus, red dots far away from nuclei and colocalizing with faint fluorescent staining may be interpreted as nerve localization of A_2A_R–D_2_R heterodimers.

Typically, A_2A_Rs are coupled to G_s_ protein, whose activation increases the cAMP levels, promoting an excitatory behavior (Weaver, 1993; Lahiri et al., 2007; De Caro et al., 2013). In the CB, the increase of adenosine levels determined by hypoxia induces the increase in intracellular cAMP through stimulation of A_2A_R (Lahiri et al., 2007). Adenosine activation of A_2A_R also triggers Ca^2+^ rise during hypoxia (Kobayashi et al., 2000; Tse et al., 2012). Similarly, A_2A_Rs also mediate the effect of hypercapnia (Sacramento et al., 2018). Conversely, D_2_Rs are coupled to inhibitory G_1_/G_0_ proteins and their activation decreases cAMP levels with the onset of an autocrine/paracrine inhibitory signal (Zeng et al., 2007; Wakai et al., 2015; Zhang et al., 2018). Hypoxia is also responsible for dopamine release from CB type I cells and activation of postsynaptic D_2_R (Prieto-Lloret et al., 2007) and D_2_R activation exerting inhibitory effects (Gonzalez et al., 1994) on ventilation, both during rest (Zapata and Zuazo, 1980) and hypoxic exposure (Nishino and Lahiri, 1981), although direct activation of D_2_R in PG terminals could also have a modulatory (Alcayaga et al., 1999; Alcayaga et al., 2003) or even excitatory effect (Alcayaga et al., 2006; Iturriaga et al., 2009), depending on species involved.

Another possible regulative mechanism exists for these receptors, based on direct reciprocal interactions. The formation of A_2A_R–D_2_R complexes was highlighted in transfected cells, including SH-SY5Y (Hillion et al., 2002; Xie et al., 2010) and HEK-293T cells (Navarro et al., 2014), neuronal primary cultures of rat striatum (Navarro et al., 2014) and enkephalin-containing GABAergic neurons from the mammal striatum (Fink et al., 1992; Fuxe et al., 1998; Trifilieff et al., 2011). A_2A_R–D_2_R heterodimers are key modulators of striatal neuronal function (Taura et al., 2018); here, heterodimerization showed to modulate GABAergic striato-pallidal neuronal activity. Reciprocal antagonistic interactions occur within the A_2A_R–D_2_R heterodimer (Fuxe et al., 2005). In particular, A_2A_R ligands decrease both the affinity and the signal intensity of D_2_R ligands (Ferré et al., 2016), determining the increased excitatory activity of adenosine, while D_2_R agonists decrease the binding of A_2A_R ligands (Fernández-Dueñas et al., 2018), causing increased inhibitory activity of dopamine. For instance, after incubation of striatal membrane preparations with the A_2A_R agonist CGS21680, the affinity of the high-affinity D_2_R agonist-binding site decreases (Fuxe et al., 1998; Guidolin et al., 2018). A_2A_R–D_2_R interactions may be modulated by different drugs (some of which with well-known effects on the CB); for instance, the psychostimulant effects of caffeine are also mediated by the blockage of the allosteric modulation within the A_2A_R–D_2_R heterodimer, by which adenosine decreases the affinity and intrinsic efficacy of dopamine at the D_2_R (Bonaventura et al., 2015).

Existence of RRI (A_2B_–D_2_) in CB chemoreceptors was first postulated by Conde et al. (2008) to explain the possible mechanism involved in catecholamine release by the CB. Thereafter, due to the glomic expression of a huge amount of different G protein-coupled receptors, our group hypothesized a possible heterodimerization between many other different receptors in the CB (Porzionato et al., 2018). Thus, the aim of this experimental work was to verify the existence of RRIs in the CB, suggesting a possible experimental strategy for its future characterization but also a new interpretive key for a broad comprehension of the regulative mechanisms it presides over.

To date, many biochemical and/or biophysical methods are available to demonstrate receptor colocalization. Among them, the PLA technique allows easy visualization of endogenous protein–protein interactions at the single molecule level (Ristic et al., 2016). Our data by PLA analysis confirmed the existence of A_2A_R–D_2_R heterodimers in both type I and type II cells of the CB, indicating that RRI may have a role in the functional modulation of these cells.

The identification of A_2A_R–D_2_R RRI in type II cells further supports a role for these cells in chemosensory modulation, in accordance with other authors (Kaelin-Lang et al., 1998; Tse et al., 2012; Leonard and Nurse, 2020). This finding, to be further detailed, could be particularly intriguing as A_2A_R–D_2_R heterodimers have also been identified in astrocytes (Cervetto et al., 2017, 2018; Pelassa et al., 2019; Guidolin et al., 2020).

The confirmation of A_2A_R–D_2_R RRI across species strengthens the idea on their contributory role in physiological events mediated by the CB. The differences between rats and humans in terms of amount and distribution of A_2A_R–D_2_R RRI may conversely be ascribed to species-specific differences and/or to potential exposure to different stimuli. The CB is known to undergo plastic changes in response to development/aging and various environmental stimuli, including chronic intermittent/sustained hypoxia. Its function is strictly related to these dynamic modifications (Iturriaga et al., 2006; López-Barneo et al., 2009; Dmitrieff et al., 2011; Kumar and Prabhakar, 2012; Bavis et al., 2013; Del Rio et al., 2014; Pulgar-Sepúlveda et al., 2018; Bavis et al., 2019; Liu et al., 2019), which can be also ascribed to the specific receptor behavior.

Various environmental stimuli could potentially modulate A_2A_R–D_2_R RRI. For instance, A_2A_R can be present intracellularly and migrate to the cell membrane upon stimulation (Arslan et al., 2002; Milojević et al., 2006; Yu et al., 2006; Sacramento et al., 2015). In this sense, hypoxic effects on RRI will be surely to be evaluated, as hypoxia exerts an increase in adenosine and dopamine release from CB chemoreceptors (Conde et al., 2012), likely inducing a receptor-level modulation, as shown in rabbits by Bairam et al. (2003). Chronic caffeine treatment induces an increase in both adenosine and dopamine (Conde et al., 2012), and neonatal caffeine treatment increases the mRNA levels encoding for A_2A_R (Montandon et al., 2008; Bairam et al., 2009) and D_2_ in male rats CB (not in female) (Montandon et al., 2008). Moreover, A_2A_R and D_2_R expressions are also modulated by age. D_2_R mRNA increases with maturation (Gauda et al., 2000, 2001; Gauda, 2000; Gauda and Lawson, 2000), whereas A_2A_R mRNA decreases (Gauda, 2000; Gauda and Lawson, 2000). Thus, further analyses will also have to address possible changes in A_2A_R–D_2_R RRI in response to development/age, hypoxic stimuli, or possible effects by other factors (drugs, metabolism, and others), allowing for a broad comparative study in different pathophysiological conditions.

Moreover, future perspectives of the work will include the involvement of other methods to better detail RRI, such as biophysical (e.g., bioluminescence– and fluorescence–resonance energy transfer; specialized microscopic techniques; X-ray crystallography) and biochemical analyses.

## Data Availability Statement

The original contributions presented in the study are included in the article/supplementary material, further inquiries can be directed to the corresponding author/s.

## Ethics Statement

The studies involving human participants were reviewed and approved by the Body Donation Program of the Section of Human Anatomy, University of Padova, according to European, Italian and regional guidelines. Excision was furtherly authorized by the Italian law No. 10 of February 10, 2020, entitled “Rules regarding the disposition of one’s body and post-mortem tissues for study, training, and scientific research purposes.” The patients/participants provided their written informed consent to participate in this study. The animal study was reviewed and approved by the Ethical Committee of Padua University, in accordance with the Italian Department of Health Guidelines.

## Author Contributions

AP, RD, VM, ES, and MS designed the study. MS and ES performed the experiments. MC and SB supported the experiments. GB and FR performed the confocal microscopy analyses. AP, RD, ES, MS, DG, and VM interpreted the data. ES and AP wrote the manuscript. RD and AP supervised the final manuscript. All authors have read and agreed to the published version of the manuscript.

## Conflict of Interest

The authors declare that the research was conducted in the absence of any commercial or financial relationships that could be construed as a potential conflict of interest.

## References

[B1] Alcayaga J. Retamal M. Cerpa V. Arroyo J. Zapata P. (2003). Dopamine inhibits ATP-induced responses in the cat petrosal ganglion in vitro. *Brain Res.* 966 283–287. 10.1016/s0006-8993(02)04215-4 12618351

[B2] Alcayaga J. Soto C. R. Vargas R. V. Ortiz F. C. Arroyo J. Iturriaga R. (2006). Carotid body transmitters actions on rabbit petrosal ganglion in vitro. *Adv. Exp. Med. Biol.* 580 331–337. 10.1007/0-387-31311-7_51 16683740

[B3] Alcayaga J. Varas R. Arroyo J. Iturriaga R. Zapata P. (1999). Dopamine modulates carotid nerve responses induced by acetylcholine on the cat petrosal ganglion in vitro. *Brain Res.* 831 97–103. 10.1016/s0006-8993(99)01402-x 10411987

[B4] Aldossary H. S. Alzahrani A. A. Nathanael D. Alhuthail E. A. Ray C. J. Batis N. (2020). G-Protein-Coupled Receptor (GPCR) Signaling in the Carotid Body: roles in Hypoxia and Cardiovascular and Respiratory Disease. *Int. J. Mol. Sci.* 21:6012. 10.3390/ijms21176012 32825527 PMC7503665

[B5] Arslan G. Kull B. Fredholm B. B. (2002). Anoxia redistributes adenosine A(2A) receptors in PC12 cells and increases receptor-mediated formation of cAMP. *Naunyn Schmiedebergs Arch. Pharmacol.* 365 150–157. 10.1007/s002100100456 11819033

[B6] Bairam A. Carroll J. L. Labelle Y. Khandjian E. W. (2003). Differential changes in dopamine D2- and D1-receptor mRNA levels induced by hypoxia in the arterial chemoreflex pathway organs in one-day-old and adult rabbits. *Biol. Neonate* 84 222–231. 10.1159/000072306 14504446

[B7] Bairam A. Dauphin C. Rousseau F. Khandjian E. W. (1996a). Dopamine D2 receptor mRNA isoforms expression in the carotid body and petrosal ganglion of developing rabbits. *Adv. Exp. Med. Biol.* 410 285–289. 10.1007/978-1-4615-5891-0_43 9030313

[B8] Bairam A. Dauphin C. Rousseau F. Khandjian E. W. (1996b). Expression of dopamine D2-receptor mRNA isoforms at the peripheral chemoreflex afferent pathway in developing rabbits. *Am. J. Respir. Cell Mol. Biol.* 15 374–381. 10.1165/ajrcmb.15.3.8810642 8810642

[B9] Bairam A. Joseph V. Lajeunesse Y. Kinkead R. (2009). Altered expression of adenosine A1 and A2A receptors in the carotid body and nucleus tractus solitarius of adult male and female rats following neonatal caffeine treatment. *Brain Res.* 1287 74–83. 10.1016/j.brainres.2009.06.064 19563784

[B10] Bairam A. Khandjian E. W. (1997). Expression of dopamine D2 receptor mRNA isoforms in the carotid body of rat, cat and rabbit. *Brain Res.* 760 287–289. 9237549 10.1016/s0006-8993(97)00399-5

[B11] Bavis R. W. Fallon S. C. Dmitrieff E. F. (2013). Chronic hyperoxia and the development of the carotid body. *Respir. Physiol. Neurobiol.* 185 94–104. 10.1016/j.resp.2012.05.019 22640932 PMC3448014

[B12] Bavis R. W. Millström A. H. Kim S. M. MacDonald C. A. O’Toole C. A. Asklof K. (2019). Combined effects of intermittent hyperoxia and intermittent hypercapnic hypoxia on respiratory control in neonatal rats. *Respir. Physiol. Neurobiol.* 260 70–81. 10.1016/j.resp.2018.11.002 30439529 PMC6326848

[B13] Bonaventura J. Navarro G. Casadó-Anguera V. Azdad K. Rea W. Moreno E. (2015). Allosteric interactions between agonists and antagonists within the adenosine A_2A_ receptor-dopamine D_2_ receptor heterotetramer. *Proc. Natl. Acad. Sci. U. S. A.* 112 E3609–E3618. 10.1073/pnas.1507704112 26100888 PMC4500251

[B14] Boscolo-Berto R. Porzionato A. Stecco C. Macchi V. De Caro R. (2020). Body donation in Italy: lights and shadows of law No. 10/2020. *Clin. Anat.* 33 950–959. 10.1002/ca.23623 32427400

[B15] Cervetto C. Venturini A. Guidolin D. Maura G. Passalacqua M. Tacchetti C. (2018). Homocysteine and A2A-D2 Receptor-Receptor Interaction at Striatal Astrocyte Processes. *J. Mol. Neurosci.* 65 456–466. 10.1007/s12031-018-1120-4 30030763

[B16] Cervetto C. Venturini A. Passalacqua M. Guidolin D. Genedani S. Fuxe K. (2017). A2A-D2 receptor-receptor interaction modulates gliotransmitter release from striatal astrocyte processes. *J. Neurochem.* 140 268–279. 10.1111/jnc.13885 27896809

[B17] Conde S. V. Gonzalez C. Batuca J. R. Monteiro E. C. Obeso A. (2008). An antagonistic interaction between A2B adenosine and D2 dopamine receptors modulates the function of rat carotid body chemoreceptor cells. *J. Neurochem.* 107 1369–1381. 10.1111/j.1471-4159.2008.05704.x 18823369

[B18] Conde S. V. Monteiro E. C. Sacramento J. F. (2017). Purines and Carotid Body: new Roles in Pathological Conditions. *Front. Pharmacol.* 8:913. 10.3389/fphar.2017.00913 29311923 PMC5733106

[B19] Conde S. V. Obeso A. Vicario I. Rigual R. Rocher A. Gonzalez C. (2006). Caffeine inhibition of rat carotid body chemoreceptors is mediated by A2A and A2B adenosine receptors. *J. Neurochem.* 98 616–628. 10.1111/j.1471-4159.2006.03912.x 16805851

[B20] Conde S. V. Ribeiro M. J. Obeso A. Rigual R. Monteiro E. C. Gonzalez C. (2012). Chronic caffeine intake in adult rat inhibits carotid body sensitization produced by chronic sustained hypoxia but maintains intact chemoreflex output. *Mol. Pharmacol.* 82 1056–1065. 10.1124/mol.112.081216 22930709

[B21] Conde S. V. Sacramento J. F. Guarino M. P. (2018). Carotid body: a metabolic sensor implicated in insulin resistance. *Physiol. Genomics.* 50 208–214. 10.1152/physiolgenomics.00121.2017 29373079

[B22] Cunha-Guimaraes J. P. Guarino M. P. Timóteo A. T. Caires I. Sacramento J. F. Ribeiro M. J. (2020). Carotid body chemosensitivity: early biomarker of dysmetabolism in humans. *Eur. J. Endocrinol.* 182 549–557. 10.1530/EJE-19-0976 32213652

[B23] Czyzyk-Krzeska M. F. Lawson E. E. Millhorn D. E. (1992). Expression of D_2_ dopamine receptor mRNA in the arterial chemoreceptor afferent pathway. *J. Auton. Nerv. Syst.* 41 31–39. 10.1016/0165-1838(92)90124-y 1362730

[B24] De Caro R. Macchi V. Porzionato A. (2009). Promotion of body donation and use of cadavers in anatomical education at the University of Padova. *Anat. Sci. Educ.* 2 91–92. 10.1002/ase.69 19215061

[B25] De Caro R. Macchi V. Sfriso M. M. Porzionato A. (2013). Structural and neurochemical changes in the maturation of the carotid body. *Respir. Physiol. Neurobiol.* 185 9–19. 10.1016/j.resp.2012.06.012 22728582

[B26] Del Rio R. Moya E. A. Iturriaga R. (2014). Carotid body potentiation during chronic intermittent hypoxia: implication for hypertension. *Front. Physiol.* 5:434. 10.3389/fphys.2014.00434 25429271 PMC4228839

[B27] Di Giulio C. (2018). Ageing of the carotid body. *J. Physiol.* 596 3021–3027. 10.1113/JP275300 29319194 PMC6068111

[B28] Dmitrieff E. F. Wilson J. T. Dunmire K. B. Bavis R. W. (2011). Chronic hyperoxia alters the expression of neurotrophic factors in the carotid body of neonatal rats. *Respir. Physiol. Neurobiol.* 175 220–227. 10.1016/j.resp.2010.11.007 21094282 PMC3033470

[B29] Fagerlund M. J. Kåhlin J. Ebberyd A. Schulte G. Mkrtchian S. Eriksson L. I. (2010). The human carotid body: expression of oxygen sensing and signaling genes of relevance for anesthesia. *Anesthesiology* 113 1270–1279. 10.1097/ALN.0b013e3181fac061 20980909

[B30] Fernández-Dueñas V. Ferré S. Ciruela F. (2018). Adenosine A2A-dopamine D2 receptor heteromers operate striatal function: impact on Parkinson’s disease pharmacotherapeutics. *Neural Regen. Res.* 13 241–243. 10.4103/1673-5374.226388 29557372 PMC5879894

[B31] Ferré S. Bonaventura J. Tomasi D. Navarro G. Moreno E. Cortés A. (2016). Allosteric mechanisms within the adenosine A2A-dopamine D2 receptor heterotetramer. *Neuropharmacology* 104 154–160. 10.1016/j.neuropharm.2015.05.028 26051403 PMC5754196

[B32] Ferré S. Casado V. Devi L. A. Filizola M. Jockers R. Lohse M. J. (2014). G protein-coupled receptor oligomerization revisited: functional and pharmacological perspectives. *Pharmacol. Rev.* 66 413–434. 10.1124/pr.113.008052 24515647 PMC3973609

[B33] Fink J. S. Weaver D. R. Rivkees S. A. Peterfreund R. A. Pollack A. E. Adler E. M. (1992). Molecular cloning of the rat A2 adenosine receptor: selective co-expression with D2 dopamine receptors in rat striatum. *Brain Res. Mol. Brain Res.* 14 186–195. 10.1016/0169-328x(92)90173-9 1279342

[B34] Fitzgerald R. S. Eyzaguirre C. Zapata P. (2009). Fifty years of progress in carotid body physiology – invited article. *Adv. Exp. Med. Biol.* 648 19–28. 10.1007/978-90-481-2259-2_2 19536461

[B35] Fuxe K. Ferré S. Canals M. Torvinen M. Terasmaa A. Marcellino D. (2005). Adenosine A2A and dopamine D2 heteromeric receptor complexes and their function. *J. Mol. Neurosci.* 26 209–219. 10.1385/JMN:26:2-3:209 16012194

[B36] Fuxe K. Ferré S. Zoli M. Agnati L. F. I. (1998). Integrated events in central dopamine transmission as analyzed at multiple levels. Evidence for intramembrane adenosine A2A/dopamine D2 and adenosine A1/dopamine D1 receptor interactions in the basal ganglia. *Brain Res. Rev.* 26 258–273. 10.1016/s0165-0173(97)00049-0 9651540

[B37] Gauda E. B. (2000). Expression and localization of A2a and A1-adenosine receptor genes in the rat carotid body and petrosal ganglia. A2a and A1-adenosine receptor mRNAs in the rat carotid body. *Adv. Exp. Med. Biol.* 475 549–558. 10849695

[B38] Gauda E. B. Bamford O. Gerfen C. R. (1996). Developmental expression of tyrosine hydroxylase, D2-dopamine receptor and substance P genes in the carotid body of the rat. *Neuroscience* 75 969–977. 10.1016/0306-4522(96)00312-0 8951888

[B39] Gauda E. B. Cooper R. Akins P. K. Wu G. (2001). Prenatal nicotine affects catecholamine gene expression in newborn rat carotid body and petrosal ganglion. *J. Appl. Physiol.* 91 2157–2165. 10.1152/jappl.2001.91.5.2157 11641357

[B40] Gauda E. B. Lawson E. E. (2000). Developmental influences on carotid body responses to hypoxia. *Respir. Physiol.* 121 199–208. 10.1016/s0034-5687(00)00128-6 10963775

[B41] Gauda E. B. Northington F. J. Linden J. Rosin D. L. (2000). Differential expression of a(2a), A(1)-adenosine and D(2)-dopamine receptor genes in rat peripheral arterial chemoreceptors during postnatal development. *Brain Res.* 872 1–10. 10.1016/s0006-8993(00)02314-3 10924669

[B42] Gonzalez C. Almaraz L. Obeso A. Rigual R. (1994). Carotid body chemoreceptors: from natural stimuli to sensory discharges. *Physiol. Rev.* 74 829–898. 10.1152/physrev.1994.74.4.829 7938227

[B43] Guidolin D. Agnati L. F. Marcoli M. Borroto-Escuela D. Fuxe K. (2015). G-protein-coupled receptor type A heteromers as an emerging therapeutic target. *Expert Opin. Ther. Targets* 19 265–283. 10.1517/14728222.2014.981155 25381716

[B44] Guidolin D. Marcoli M. Tortorella C. Maura G. Agnati L. F. (2018). G protein-coupled receptor-receptor interactions give integrative dynamics to intercellular communication. *Rev. Neurosci.* 29 703–726. 10.1515/revneuro-2017-0087 29466243

[B45] Guidolin D. Marcoli M. Tortorella C. Maura G. Agnati L. F. (2020). Adenosine A2A-dopamine D2 receptor-receptor interaction in neurons and astrocytes: evidence and perspectives. *Prog. Med. Biol. Transl. Sci.* 169 247–277. 10.1016/bs.pmbts.2019.11.004 31952688

[B46] Hillion J. Canals M. Torvinen M. Casadó V. Scott R. Terasmaa A. (2002). Coaggregation, cointernalization, and codesensitization of adenosine A2A receptors and dopamine D2 receptors. *J. Biol. Chem.* 277 18091–18097. 10.1074/jbc.M107731200 11872740

[B47] Holgert H. Hökfelt T. Hertzberg T. Lagercrantz H. (1995). Functional and developmental studies of the peripheral arterial chemoreceptors in rat: effects of nicotine and possible relation to sudden infant death syndrome. *Proc. Natl. Acad. Sci. U. S. A.* 92 7575–7579. 10.1073/pnas.92.16.7575 7638233 PMC41382

[B48] Iturriaga R. (2018). Translating carotid body function into clinical medicine. *J. Physiol.* 596 3067–3077. 10.1113/JP275335 29114876 PMC6068206

[B49] Iturriaga R. Alcayaga J. (2004). Neurotransmission in the carotid body: transmitters and modulators between glomus cells and petrosal ganglion nerve terminals. *Brain Res. Brain Res. Rev.* 47 46–53. 10.1016/j.brainresrev.2004.05.007 15572162

[B50] Iturriaga R. Alcayaga J. Gonzalez C. (2009). Neurotransmitters in carotid body function: the case of dopamine–invited article. *Adv. Exp. Med. Biol.* 648 137–143. 10.1007/978-90-481-2259-2_16 19536475

[B51] Iturriaga R. Del Rio R. Idiaquez J. Somers V. K. (2016). Carotid body chemoreceptors, sympathetic neural activation, and cardiometabolic disease. *Biol. Res.* 49:13. 10.1186/s40659-016-0073-8 26920146 PMC4768417

[B52] Iturriaga R. Rey S. Alcayaga J. Del Rio R. (2006). Chronic intermittent hypoxia enhances carotid body chemosensory responses to acute hypoxia. *Adv. Exp. Med. Biol.* 580 227–232. 10.1007/0-387-31311-7_35 16683724

[B53] Kaelin-Lang A. Lauterburg T. Burgunder J. M. (1998). Expression of adenosine A2a receptor gene in rat dorsal root and autonomic ganglia. *Neurosci. Lett.* 246 21–24. 10.1016/s0304-3940(98)00216-x 9622198

[B54] Katz D. M. Black I. B. (1986). Expression and regulation of catecholaminergic traits in primary sensory neurons: relationship to target innervation in vivo. *J. Neurosci.* 6 983–989. 10.1523/JNEUROSCI.06-04-00983.1986 2422331 PMC6568438

[B55] Katz D. M. Markey K. A. Goldstein M. Black I. B. (1983). Expression of catecholaminergic characteristics by primary sensory neurons in the normal adult rat in vivo. *Proc. Natl. Acad. Sci. U. S. A.* 80 3526–3530. 10.1073/pnas.80.11.3526 6134285 PMC394078

[B56] Kinkead R. Joseph V. Lajeunesse Y. Bairam A. (2005). Neonatal maternal separation enhances dopamine D(2)-receptor and tyrosine hydroxylase mRNA expression levels in carotid body of rats. *Can. J. Physiol. Pharmacol.* 83 76–84. 10.1139/y04-106 15759053

[B57] Kobayashi S. Conforti L. Millhorn D. E. (2000). Gene expression and function of adenosine A(2A) receptor in the rat carotid body. *Am. J. Physiol. Lung Cell. Mol. Physiol.* 279 L273–L282. 10.1152/ajplung.2000.279.2.L273 10926550

[B58] Kumar P. Prabhakar N. R. (2012). Peripheral chemoreceptors: function and plasticity of the carotid body. *Compr. Physiol.* 2 141–219. 10.1002/cphy.c100069 23728973 PMC3919066

[B59] Lahiri S. Mitchell C. H. Reigada D. Roy A. Cherniack N. S. (2007). Purines, the carotid body and respiration. *Respir. Physiol. Neurobiol.* 157 123–129. 10.1016/j.resp.2007.02.015 17383945 PMC1975770

[B60] Leonard E. M. Nurse C. A. (2020). Expanding Role of Dopaminergic Inhibition in Hypercapnic Responses of Cultured Rat Carotid Body Cells: involvement of Type II Glial Cells. *Int. J. Mol. Sci.* 21:5434. 10.3390/ijms21155434 32751703 PMC7432366

[B61] Liu P. Zhang H. M. Hu K. Zhou X. F. Tang S. (2019). Sensory plasticity of carotid body is correlated with oxidative stress in paraventricular nucleus during chronic intermittent hypoxia. *J. Cell Physiol.* 234 13534–13543. 10.1002/jcp.28031 30609027 PMC13483341

[B62] López-Barneo J. Ortega-Sáenz P. Pardal R. Pascual A. Piruat J. I. Durán R. (2009). Oxygen sensing in the carotid body. *Ann. N. Y. Acad. Sci.* 1177 119–131. 10.1111/j.1749-6632.2009.05033.x 19845614

[B63] Macchi V. Porzionato A. Stecco C. Tiengo C. Parenti A. Cestrone A. (2011). Body parts removed during surgery: a useful training source. *Anat. Sci. Educ.* 4 151–156. 10.1002/ase.218 21491611

[B64] Michel M. C. Wieland T. Tsujimoto G. (2009). How reliable are G-protein-coupled receptor antibodies? *Naunyn Schmiedebergs Arch. Pharmacol.* 379 385–388. 10.1007/s00210-009-0395-y 19172248

[B65] Milojević T. Reiterer V. Stefan E. Korkhov V. M. Dorostkar M. M. Ducza E. (2006). The ubiquitin-specific protease Usp4 regulates the cell surface level of the A2A receptor. *Mol. Pharmacol.* 69 1083–1094. 10.1124/mol.105.015818 16339847

[B66] Montandon G. Bairam A. Kinkead R. (2008). Neonatal caffeine induces sex-specific developmental plasticity of the hypoxic respiratory chemoreflex in adult rats. *Am. J. Physiol. Regul. Integr. Comp. Physiol.* 295 R922–R934. 10.1152/ajpregu.00059.2008 18596110

[B67] Navarro G. Aguinaga D. Moreno E. Hradsky J. Reddy P. P. Cortés A. (2014). Intracellular calcium levels determine differential modulation of allosteric interactions within G protein-coupled receptor heteromers. *Chem. Biol.* 21 1546–1566. 10.1016/j.chembiol.2014.10.004 25457181 PMC9875831

[B68] Nishino T. Lahiri S. (1981). Effects of dopamine on chemoreflexes in breathing. *J. Appl. Physiol.* 50 892–897. 10.1152/jappl.1981.50.4.892 6790490

[B69] Nurse C. A. (2005). Neurotransmission and neuromodulation in the chemosensory carotid body. *Auton. Neurosci.* 120 1–9. 10.1016/j.autneu.2005.04.008 15955746

[B70] Nurse C. A. (2014). Synaptic and paracrine mechanisms at carotid body arterial chemoreceptors. *J. Physiol.* 592 3419–3426. 10.1113/jphysiol.2013.269829 24665097 PMC4229340

[B71] Pardal R. Ortega-Sáenz P. Durán R. López-Barneo J. (2007). Glia-like stem cells sustain physiologic neurogenesis in the adult mammalian carotid body. *Cell* 131 364–377. 10.1016/j.cell.2007.07.043 17956736

[B72] Pelassa S. Guidolin D. Venturini A. Averna M. Frumento G. Campanini L. (2019). A2A-D2 Heteromers on Striatal Astrocytes: biochemical and Biophysical Evidence. *Int. J. Mol. Sci.* 20:2457. 10.3390/ijms20102457 31109007 PMC6566402

[B73] Porzionato A. Macchi V. Belloni A. S. Parenti A. De Caro R. (2006). Adrenomedullin immunoreactivity in the human carotid body. *Peptides* 27 69–73. 10.1016/j.peptides.2005.07.017 16154664

[B74] Porzionato A. Macchi V. Guidolin D. Parenti A. Ferrara S. D. De Caro R. (2005). Histopathology of carotid body in heroin addiction. Possible chemosensitive impairment. *Histopathology* 46 296–306. 10.1111/j.1365-2559.2005.02060 15720415

[B75] Porzionato A. Macchi V. Parenti A. De Caro R. (2008). Trophic factors in the carotid body. *Int. Rev. Cell Mol. Biol.* 269 1–58. 10.1016/S1937-6448(08)01001-0 18779056

[B76] Porzionato A. Macchi V. Stecco C. Mazzi A. Rambaldo A. Sarasin G. (2012). Quality management of Body Donation Program at the University of Padova. *Anat. Sci. Educ.* 5 264–272. 10.1002/ase.1285 22573575

[B77] Porzionato A. Rucinski M. Macchi V. Stecco C. Castagliuolo I. Malendowicz L. K. (2011). Expression of leptin and leptin receptor isoforms in the rat and human carotid body. *Brain Res.* 1385 56–67. 10.1016/j.brainres.2011.02.028 21334312

[B78] Porzionato A. Stocco E. Guidolin D. Agnati L. Macchi V. De Caro R. (2018). Receptor-Receptor Interactions of G Protein-Coupled Receptors in the Carotid Body: a Working Hypothesis. *Front. Physiol.* 9:697. 10.3389/fphys.2018.00697 29930516 PMC6000251

[B79] Prabhakar N. R. Peng Y. J. (2017). Oxygen Sensing by the Carotid Body: past and Present. *Adv. Exp. Med. Biol.* 977 3–8. 10.1007/978-3-319-55231-6_1 28685420

[B80] Prieto-Lloret J. Donnelly D. F. Rico A. J. Moratalla R. González C. Rigual R. J. (2007). Hypoxia transduction by carotid body chemoreceptors in mice lacking dopamine D(2) receptors. *J. Appl. Physiol.* 103 1269–1275. 10.1152/japplphysiol.00391.2007 17673562

[B81] Pulgar-Sepúlveda R. Varas R. Iturriaga R. Del Rio R. Ortiz F. C. (2018). Carotid Body Type-I Cells Under Chronic Sustained Hypoxia: focus on Metabolism and Membrane Excitability. *Front. Physiol.* 9:1282. 10.3389/fphys.2018.01282 30283346 PMC6157308

[B82] Riederer B. M. Bolt S. Brenner E. Bueno-Lopez J. L. Circulescu A. R. M. Davies D. C. (2012). The legal and ethical framework governing Body Donation in Europe–1st update on current practice. *Eur. J. Anat.* 16 1–21.

[B83] Ristic M. Brockly F. Piechaczyk M. Bossis G. (2016). Detection of Protein–Protein Interactions and Posttranslational Modifications Using the Proximity Ligation Assay: application to the Study of the SUMO Pathway. *Methods Mol. Biol.* 1449 279–290. 10.1007/978-1-4939-3756-1_17 27613043

[B84] Sacramento J. F. Andrzejewski K. Melo B. F. Ribeiro M. J. Obeso A. Conde S. V. (2020). Exploring the Mediators that Promote Carotid Body Dysfunction in Type 2 Diabetes and Obesity Related Syndromes. *Int. J. Mol. Sci.* 21:5545. 10.3390/ijms21155545 32756352 PMC7432672

[B85] Sacramento J. F. Melo B. F. Conde S. V. (2018). Adenosine Mediates Hypercapnic Response in the Rat Carotid Body via A2A and A2B Receptors. *Adv. Exp. Med. Biol.* 1071 89–93. 10.1007/978-3-319-91137-3_11 30357738

[B86] Sacramento J. F. Olea E. Ribeiro M. J. Prieto-Lloret J. Melo B. F. Gonzalez C. (2019). Contribution of adenosine and ATP to the carotid body chemosensory activity in ageing. *J. Physiol.* 597 4991–5008. 10.1113/JP274179 31426127

[B87] Sacramento J. F. Ribeiro M. J. Yubero S. Melo B. F. Obeso A. Guarino M. P. (2015). Disclosing caffeine action on insulin sensitivity: effects on rat skeletal muscle. *Eur. J. Pharm. Sci.* 70 107–116. 10.1016/j.ejps.2015.01.011 25661425

[B88] Schamel A. Verna A. (1993). Localization of dopamine D2 receptor mRNA in the rabbit carotid body and petrosal ganglion by in situ hybridization. *Adv. Exp. Med. Biol.* 337 85–91. 10.1007/978-1-4615-2966-8_13 8109435

[B89] Stocco E. Barbon S. Tortorella C. Macchi V. De Caro R. Porzionato A. (2020). Growth Factors in the Carotid Body-An Update. *Int. J. Mol. Sci.* 21:7267. 10.3390/ijms21197267 33019660 PMC7594035

[B90] Taura J. Valle-León M. Sahlholm K. Watanabe M. Van Craenenbroeck K. Fernández-Dueñas V. (2018). Behavioral control by striatal adenosine A2A -dopamine D2 receptor heteromers. *Genes Brain Behav.* 17:e12432. 10.1111/gbb.12432 29053217

[B91] Trifilieff P. Rives M. L. Urizar E. Piskorowski R. A. Vishwasrao H. D. Castrillon J. (2011). Detection of antigen interactions ex vivo by proximity ligation assay: endogenous dopamine D2-adenosine A2A receptor complexes in the striatum. *Biotechniques* 51 111–118. 10.2144/000113719 21806555 PMC3642203

[B92] Tse A. Yan L. Lee A. K. Tse F. W. (2012). Autocrine and paracrine actions of ATP in rat carotid body. *Can. J. Physiol. Pharmacol.* 90 705–711. 10.1139/y2012-054 22509744

[B93] Varas R. Alcayaga J. Iturriaga R. (2003). ACh and ATP mediate excitatory transmission in cat carotid identified chemoreceptor units in vitro. *Brain Res.* 988 154–163. 10.1016/s0006-8993(03)03366-3 14519537

[B94] Wakai J. Takayama A. Yokoyama T. Nakamuta N. Kusakabe T. Yamamoto Y. (2015). Immunohistochemical localization of dopamine D2 receptor in the rat carotid body. *Acta Histochem.* 117 784–789. 10.1016/j.acthis.2015.07.007 26272445

[B95] Weaver D. R. (1993). A2a adenosine receptor gene expression in developing rat brain. *Brain Res. Mol. Brain Res.* 20 313–327. 10.1016/0169-328x(93)90058-w 8114618

[B96] Xie H. Hu L. Li G. (2010). SH-SY5Y human neuroblastoma cell line: in vitro cell model of dopaminergic neurons in Parkinson’s disease. *Chin. Med. J.* 123 1086–1092. 20497720

[B97] Xu F. Xu J. Tse F. W. Tse A. (2006). Adenosine stimulates depolarization and rise in cytoplasmic [Ca2+] in type I cells of rat carotid bodies. *Am. J. Physiol. Cell Physiol.* 290 C1592–C1598. 10.1152/ajpcell.00546.2005 16436472

[B98] Yu W. Zacharia L. C. Jackson E. K. Apodaca G. (2006). Adenosine receptor expression and function in bladder uroepithelium. *Am. J. Physiol. Cell. Physiol.* 291 C254–C265. 10.1152/ajpcell.00025.2006 16571869

[B99] Zapata P. Zuazo A. (1980). Respiratory effects of dopamine-induced inhibition of chemosensory inflow. *Respir. Physiol.* 40 79–92. 10.1016/0034-5687(80)90006-7 6248944

[B100] Zeng C. Zhang M. Asico L. D. Eisner G. M. Jose P. A. (2007). The dopaminergic system in hypertension. *Clin. Sci.* 112 583–597. 10.1042/CS20070018 17492945

[B101] Zhang M. Vollmer C. Nurse C. A. (2018). Adenosine and dopamine oppositely modulate a hyperpolarization-activated current I h in chemosensory neurons of the rat carotid body in co-culture. *J. Physiol.* 596 3101–3117. 10.1113/JP274743 28801916 PMC6068256

